# Eph-ephrin signaling affects lens growth and shape, nucleus size, and gradient refractive index in adult mice

**DOI:** 10.3389/fopht.2025.1688964

**Published:** 2025-10-31

**Authors:** Gryffin M. Flowers, Kehao Wang, Masato Hoshino, Kentaro Uesugi, Naoto Yagi, Barbara Pierscionek, Catherine Cheng

**Affiliations:** 1 School of Optometry and Vision Science Program, Indiana University, Bloomington, IN, United States; 2 Beijing Advanced Innovation Center for Biomedical Engineering, School of Engineering Medicine, Beihang University, Beijing, China; 3 Japan Synchrotron Radiation Research Institute (SPring-8), Sayo-cho, Hyogo, Japan; 4 Faculty of Health, Medicine and Social Care, Medical Technology Research Centre, Anglia Ruskin University, Chelmsford, United Kingdom

**Keywords:** epithelial cells, fiber cells, morphometrics, strain, grin

## Abstract

**Purpose:**

The function of the eye lens, to fine focus light from different distances onto the retina to form a clear image, relies on tissue biomechanical properties, refractive index, shape, and transparency. Increased lens stiffness with age, especially of the center or nucleus, has long been hypothesized to lead to presbyopia, a loss of accommodative ability, and the need for reading glasses. The cellular and molecular mechanisms that determine lens biomechanical properties and change during age-related stiffening remain unclear. Little is known about the factors that regulate lens shape and growth, nucleus size, and refractive index. We previously showed that loss of EphA2, a receptor tyrosine kinase, or ephrin-A5, a ligand for Eph receptors, leads to changes in lens shape and resilience in 2-month-old mice. Surprisingly, the loss of EphA2 led to smaller and softer lens nuclei with no change in lens stiffness.

**Methods:**

Using coverslip compression and X-ray phase tomography, we investigated whether lens stiffness, resilience, morphometric changes, and gradient refractive index (GRIN) were altered in lenses from 4- and 8-month-old adult mice with disruption of Eph-ephrin signaling.

**Results:**

Our data revealed no obvious changes in lens stiffness or resilience between control and ephrin-A5 knockout (KO or -/-) mice at 4 and 8 months of age. While there were no differences in lens resilience, *EphA2^-/-^* lenses were stiffer than control lenses from 8-month-old mice. At all ages, EphA2 and ephrin-A5 KO lenses were more spherical in shape, and *EphA2^-/-^* lens nuclei were smaller than controls. In 4- and 8-month-old mice, *EphA2^-/-^* lenses were small. Measurement of GRIN in control and KO lenses revealed that *EphA2^-/-^* lenses had decreased magnitudes of refractive index across the GRIN profile in all age groups.

**Conclusions:**

These results suggest that, at least in mouse lenses, the size of the lens and nucleus does not affect whole tissue stiffness with age. Our work indicates that Eph-ephrin signaling influences lens shape and normal adult whole lens growth while EphA2 is needed for nuclear size and appropriate GRIN.

## 1 Introduction

The lens is a transparent, flexible, and ellipsoid tissue in the anterior chamber of the eye that is responsible for fine focusing of light onto the retina. The anterior hemisphere of the lens is covered by a single layer of epithelial cells, which are responsible for maintaining lens homeostasis and required for lifelong lens growth (1). While anterior epithelial cells are quiescent, epithelial cells grow and divide at the lens equator before differentiating into secondary lens fiber cells (**Supplementary Figure 1A**) (1, 2). These newly formed fiber cells at the lens equator elongate towards the anterior and posterior poles, and at each pole, the tips of the fiber cells will detach from the anterior epithelium or the posterior capsule to contact the tips of fiber cells elongating from other directions to meet and make a Y-suture pattern (3–5). New layers of fiber cells surround and overlay previous generations of fibers. During the maturation process for lens fiber cells, cellular organelles are degraded to allow for a clear light path (6, 7). The innermost lens fiber cells have been present since before birth, and these central fiber cells make up the lens nucleus (8–10), which approximates to the zone of highest refractive index in the lens (11–13). A thin collagenous membrane, called the lens capsule, surrounds the whole tissue (1). The mechanisms that regulate whole lens biomechanics, growth, and shape and lens nucleus size and stiffness remain unclear.

Suspended behind the iris, the lens is held in place via zonular fibers that extend from the lens capsule to the surrounding ciliary body processes (14). When the eyes look out into the distance, the ciliary muscle is relaxed, pulling the zonular fibers taut, causing the lens to be relatively flattened (14). When looking near, the ciliary muscle contracts, loosening the zonular fibers allowing the lens to relax and become more rounded (14). This flexible nature of the lens allows individuals to see clearly across many distances. However, with the normal aging process, the lens stiffens, losing the ability to change shape and to keep light finely focused on the retina for viewing near objects. This loss of focusing ability is known as presbyopia. There are treatments for presbyopia such as readers, bifocals, specialty contacts, or recently FDA-approved pupil constriction eyedrops (15–18), but there are no current methods for preventing or delaying the onset of presbyopia.

Age-related decreases in accommodative ability of human lenses are not directly related to changes of ciliary muscle action or to zonular fiber elasticity. Zonular elasticity does not change significantly with age (19, 20), and while ciliary muscle action and its connection to peripheral ocular tissues has subtle changes (19, 21, 22), the overarching cause of presbyopia appears related to lens biomechanical properties (23) and to its continuous growth (8). It has long been hypothesized that in the human eye, increased lens size and stiffness with age, in particular the lens nucleus, lead to presbyopia (8, 9, 24–28). Like human lenses, mouse lenses increase in size and stiffness with age (10, 29–32), and the rigid lens nucleus also increases in size in both species (10, 29). This makes the mouse a good model for determining potential causes of age-related stiffness changes in the lens.

Recent studies have linked Eph-ephrin signaling to lens pathologies [reviewed in (33)], including changes in biomechanical and morphometric properties (5, 11). Erythropoietin-producing hepatocellular carcinoma (Eph) receptors are receptor tyrosine kinases associated with cell-cell adhesion (34), repulsion (35), motility (36), and protrusions (11) as well as cell development and patterning (34, 37) and cell differentiation (38–40). Eph receptors bind to a class of cell surface bound ligands known as Eph receptor-interacting proteins (ephrins) (33). Ephrins help mediate functions such as cell proliferation (41), adhesion (42, 43), migration (43), and repulsion (44). Depending on the mouse strain background, loss of the receptor EphA2 or the ligand ephrin-A5 can lead to severe lens degeneration and cataracts (45, 46) or milder phenotypes including nuclear or anterior cataracts, respectively (47). Our previous studies of mice in C57BL/6J genetic background show that EphA2 is mainly expressed in the lens fibers and equator epithelial cells while ephrin-A5 is primarily found in the anterior epithelium (47, 48). EphA2 contributes to lens phenotypic changes including equatorial epithelial and fiber cell organization and hexagonal cell shape (48–50). In contrast, ephrin-A5 plays a role in maintaining the anterior epithelial monolayer (47, 49). Our previous data demonstrate that, in 2-month-old mice, loss of either EphA2 or ephrin-A5 does not alter lens stiffness when compared to respective controls (11), but knockout (KO or *^-/-^*) lenses have increased resilience (**Supplementary Figure 1B**) (5). Morphometric data shows that lenses from 2-month-old *EphA2^-/-^* and *ephrin-A5^-/-^* mice have reduced aspect ratios leading to more spherical lenses compared to their associated control groups (11). In addition, lenses from 2-month-old *EphA2^-/-^* mice display smaller nuclei and a lower gradient refractive index (GRIN) (11).

In this study, we explore morphometric and biomechanical properties of the lens to determine whether Eph-ephrin signaling contributes to age-related stiffness changes. In lenses from 4- and 8-month-old *EphA2^-/-^* and *ephrin-A5^-/-^* mice and their respective control groups, we measured morphometrics and then stiffness and resilience via sequential coverslip application within a fluid-filled chamber. These data were compared to our prior data from 2-month-old mice (5, 11), which allowed age-based analysis on factors contributing to lens biomechanical properties and morphometrics. Biomechanical testing showed lenses from 8-month-old *EphA2^-/-^* mice were stiffer than those of 8-month-old control *EphA2^+/+^* mice. We find that loss of EphA2 leads to decreased eye, lens, and nucleus volumes as well as the overall magnitude of the GRIN profile and max refractive index in young and older adult mice. Loss of either EphA2 or ephrin-A5 leads to more spherical lenses and a lack of lens growth between 2 and 4 months of age. These data indicate that the ligand ephrin-A5 does not play a significant role in lens biomechanical properties or nucleus growth and size. Overall, we show that Eph-ephrin signaling affects lens shape and postnatal lens growth and that EphA2 is required for normal GRIN and nucleus growth. Our results demonstrate that reduced lens and nucleus volumes do not prevent age-related stiffening of the whole lens.

## 2 Materials and methods

### 2.1 Animals

The care and maintenance of mice was performed in accordance with an approved Institutional Animal Care and Use Committee protocol (#24-002) and the Guide for the Care and Use of Laboratory Animals by the National Institutes of Health. *EphA2^-/-^* mice were acquired from The Jackson Laboratory (strain #: 006028), and *ephrin-A5^-/-^* mice (51) were a generous gift from Dr. David A. Feldheim (University of California, Santa Cruz, CA, USA). Both mouse lines were backcrossed onto the C57BL/6J wild-type (*WT*) background for 10–12 generations. To prevent the unknown influence of genetic drift, mating pairs of mice with heterozygous genotypes were used to produce control (*EphA2^+/+^* or *ephrin-A5^+/+^*) and KO (*EphA2^-/-^* or *ephrin-A5^-/-^*) littermates for experiments. Approximately equal numbers of male and female mice at 4 and 8 months of age were used in experiments, and we did not observe any obvious differences between the lens phenotypes of male vs. female mice. Comparison of eye volume and lens volume revealed no differences between male and female mice of the same age and genotype (data not shown). Genotyping was performed using automated qPCR on toe and/or tail snips collected between postnatal days 5-7 (Transnetyx, Cordova, TN, USA) as previously described (47), and genotyping also confirmed that all mice were maintained in the C57BL/6J background with wild-type *Bfsp2* (CP49) genes (52, 53). Previous studies showed that loss of CP49 resulting from spontaneous mutations in several mouse genetic backgrounds leads to changes in lens transparency and biomechanics (31, 54). *Ephrin-A5^-/-^* lenses can display anterior subcapsular cataracts with compromised fiber cell layers (47, 49), and those lenses were excluded from this study due to possible biomechanical defects related to fiber cell abnormalities that are secondary to epithelial cell defects.

### 2.2 Lens biomechanical testing and morphometrics

Overhead images of freshly enucleated whole eyes were captured with a Zeiss Discovery V8 dissecting microscope with digital camera (Carl Zeiss Microscopy, Jena, Germany). Biomechanics and morphometrics of freshly dissected lenses from 4- and 8-month-old *EphA2^+/+^*, *EphA2^-/-^*, *ephrin-A5^+/+^*, and *ephrin-A5^-/-^* mice were measured in 1X DPBS (14190; ThermoFisher, Carlsbad, California, USA) at room temperature as previously described (29, 31). Eight lenses from at least 4 mice were used for each age group of each genotype in these experiments. The left and right eyes of each mouse were treated as separate biological samples. Unilateral eye defects have been reported in human patients (55, 56) and mouse models with genetic defects (57, 58), suggesting that the eyes are independent samples. Within a custom designed compression chamber, lenses from 4- and 8-month-old mice were compressed in a 300µm-deep round divot. Coverslips, with an average (59) weight of 115.68mg, were placed, in sequence, onto lenses to compress the tissue. After each coverslip was added, the tissue was allowed to equilibrate for 2 minutes before images of the lens were acquired using a right-angle mirror with the dissecting microscope and digital camera. After compression testing, the lens capsule was carefully removed using sharp tweezers, and soft cortical fiber cells were removed by gently rolling the decapsulated lens between wet gloved fingertips, leaving behind the central hard and round lens nucleus for imaging (10, 29, 31). Mouse lens nuclei are very hard and are not damaged by this mechanical isolation method.

Axial and equatorial diameters of eyes and lenses were measured from pictures using the ZEN 3.6 (blue edition; Carl Zeiss Microscopy) software, and Excel and GraphPad Prism 10 (GraphPad Software, Boston, MA) were used to calculate and plot axial and equatorial strain [ϵ = (d-d_0_)/d_0_, where ϵ is strain, d is the axial or equatorial diameter at a given load, and d_0_ is the corresponding axial or equatorial diameter at zero load], lens resilience (ratio between pre- and post-load axial diameters), eye volume (volume = 4/3 x π x r_Eeye_^2^ x r_Aeye_, where r_Eeye_ is the equatorial radius of the eye and r_Aeye_ is the axial radius of the eye), lens volume (volume = 4/3 x π x r_E_^2^ x r_A_, where r_E_ is the equatorial radius and r_A_ is the axial radius), lens aspect ratio (ratio between equatorial and axial diameters), and nuclear volume (volume = 4/3 x π x r_N_^3^, where r_N_ is the radius of the lens nucleus). Plots represent the mean ± standard deviation, and data from 2-month-old mice were replotted from our previous studies (5, 11) and analyzed in comparison to results from 4- and 8-month-old mice. For morphometric and resilience comparisons between respective control and KO measurements from each age group, student *t*-test was used to determine statistical significance. When comparing morphometric and resilience data between the three age groups across the same genotype, one-way ANOVA with Tukey multiple comparison corrections were used to determine statistical significance. For strain data, two-way ANOVA test with Šidák correction for multiple comparisons was used to determine statistical significance.

### 2.3 X-ray phase tomography

X-ray phase tomography using X-ray Talbot interferometry to measure the GRIN profile was performed as previously described at the SPring-8 facility (Japan) (29, 60–62). Enucleated eyes from 4- and 8-month-old control and KO mice were collected, shipped, and stored in Dulbecco’s modified Eagle medium without phenol red (21063–029; ThermoFisher) with 2% penicillin/streptomycin (15-140-122; ThermoFisher) at room temperature before experiments. Experiments were conducted within 4 days post-mortem. The lenses were measured inside intact eyeballs, and no evidence of lens swelling was noted in any samples. Matlab (2022a; MathWorks, Natick, MA, USA) was used to calculate the refractive index from interferometry measurements to generate two-dimensional (2D) iso-indicial index contours and three-dimensional (3D) meshed index profiles in the mid-sagittal plane (anterior-posterior plane through the visual axis) of each mouse eye through the center of the lens. GRIN profiles were generated along the visual axis by Matlab, and the means and standard deviations were calculated in Excel and plotted in GraphPad Prism 10. At least 6 eyes from each genotype for all three age groups were analyzed. Data from 2-month-old mice were replotted from our previous study (11) for comparison. For *EphA2^+/+^* mice, 10 and 16 eyes were measured from 4- and 8-month-old animals, respectively. For *EphA2^-/-^* mice, 12 and 26 eyes were measured from 4- and 8-month-old animals, respectively. For *ephrin-A5^+/+^* mice, 7 and 16 eyes were measured from 4- and 8-month-old animals, respectively. For *ephrin-A5^-/-^* mice, 7 and 11 eyes were measured from 4- and 8-month-old animals, respectively. Welch’s *t*-test between KO and respective control samples were used to determine GRIN statistical significance (11). For max refractive index comparisons between respective control and KO measurements from each age group, student *t*-test was used to determine statistical significance. When comparing max refractive index between the three age groups across the same genotype, one-way ANOVA with Tukey multiple comparison corrections were used to determine statistical significance.

## 3 Results

### 3.1 Disruption of EphA2 and ephrin-A5 leads to changes in eye and lens morphometrics

**Figure 1:**
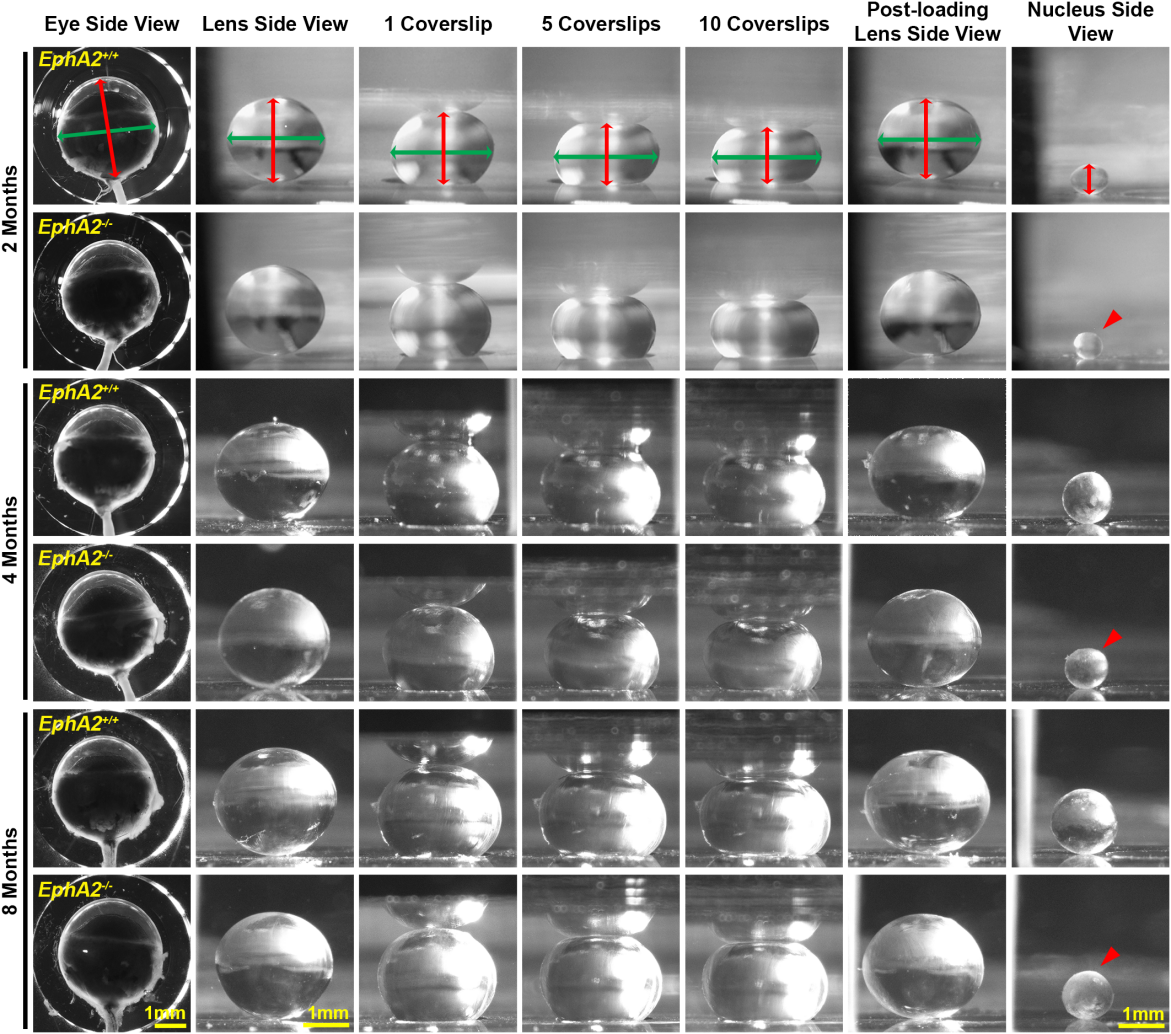
Pictures of eyes and lenses from *EphA2^+/+^* and *EphA2^-/-^* mice between 2–8 months of age. Freshly enucleated eyes were imaged before lens microdissection. Lenses were imaged pre-compression, during coverslip compression (1, 5, and 10 coverslips), and post-compression, and then the lens nucleus was isolated for imaging. Under the same load, lenses from older mice were less compressed than lenses from younger mice. With age, there is an overall increase in eye, lens, and nucleus size in both control and KO mice; however, *EphA2^-/-^* lens nuclei are smaller in all three age groups (red arrowheads). The axial diameter (red double-headed arrows) and equatorial diameter (green double-headed arrows) of each eye, lens, and lens nucleus were measured to calculate eye volume, lens volume, lens aspect ratio, axial compressive strain, equatorial expansive strain, resilience, and nuclear volume. Scale bars, 1mm.

**Figure 2:**
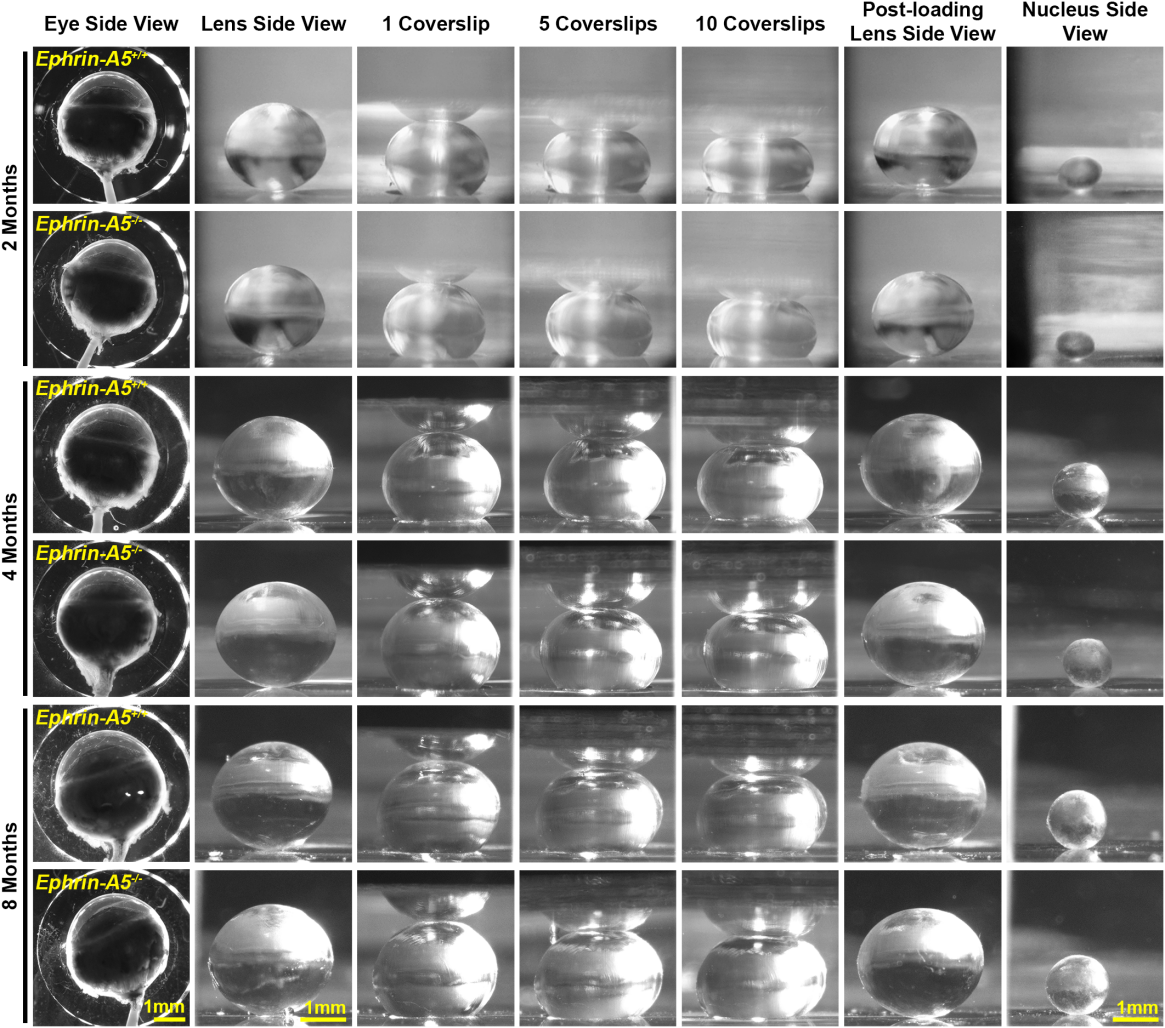
Pictures of eyes and lenses from *ephrin-A5^+/+^* and *ephrin-A5^-/-^* mice between 2–8 months of age. Freshly enucleated eyes were imaged before lens microdissection. Lenses were imaged pre-compression, during coverslip compression (1, 5, and 10 coverslips), and post-compression, and then the lens nucleus was isolated for imaging. Under the same load, lenses from older mice were less compressed than lenses from younger mice. With age, there is an overall increase in eye, lens, and nucleus size in both control and *ephrin-A5^-/-^* mice. Scale bars, 1mm.

**Figure 3:**
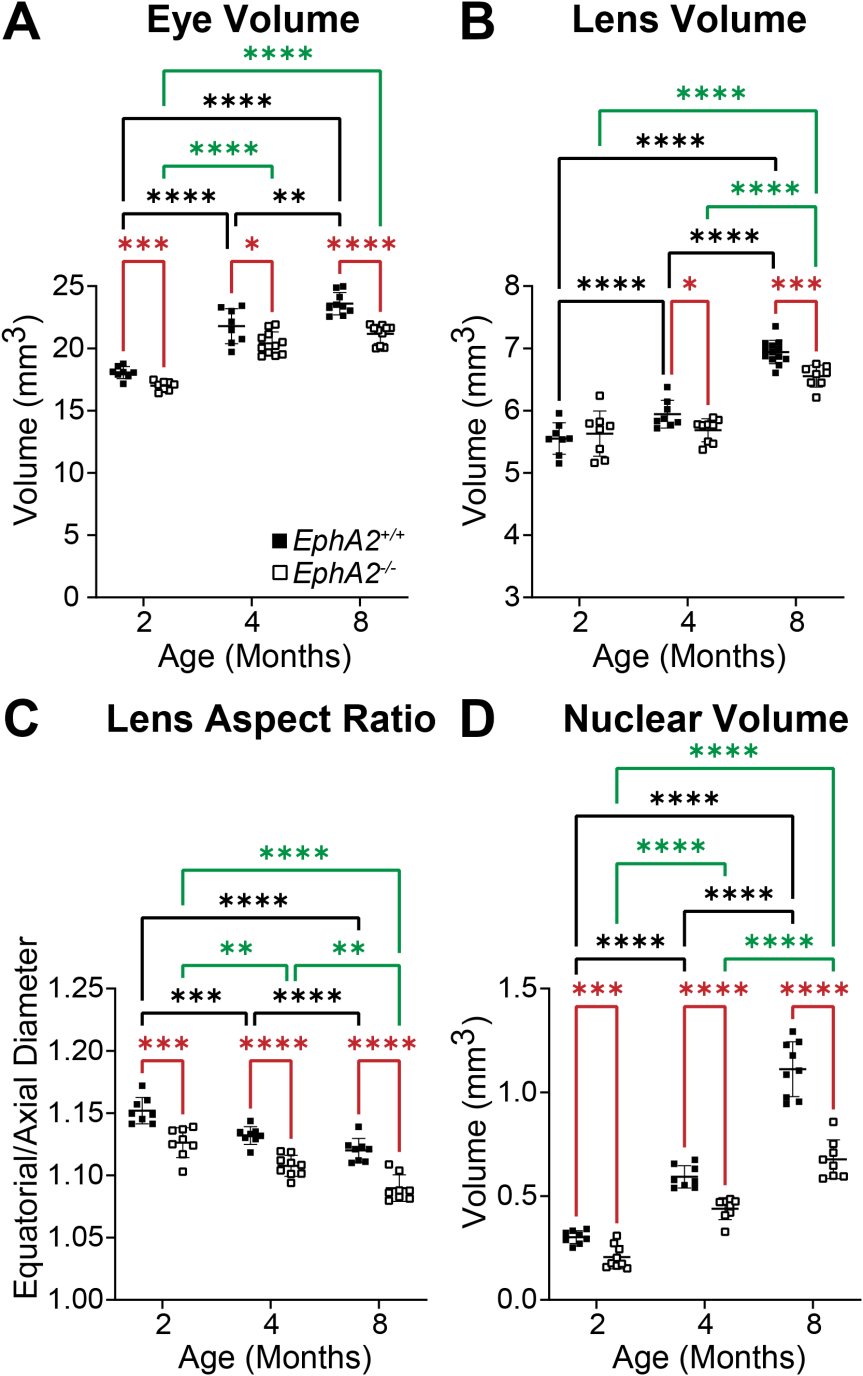
Morphometric measurements of eyes, lenses, and lens nuclei from 2-, 4-, and 8-month-old *EphA2^+/+^* and *EphA2^-/-^* mice. Data from the 2-month-old mice are replotted from our previous work (11). Data on the graphs reflect the mean ± standard deviation (SD) from at least 8 lenses per age and genotype. **(A)***EphA2^-/-^* eye volumes are smaller than controls in all age groups. In *EphA2^+/+^* mice, eye volume increases with age, while in *EphA2^-/-^* mice, eye volume increases from 2 to 4 months of age and then plateaus with no increase between 4 to 8 months of age. **(B)** While lens volume increases with age in *EphA2^+/+^* mice, *EphA2^-/-^* lens volume does not change between 2 and 4 months of age and increases between 4 and 8 months of age. Lens volume for 4- and 8-month *EphA2^-/-^* mice are lower than controls. **(C)***EphA2^-/-^* lenses are more spherical (decreased lens aspect ratio) than control lenses in all age groups. In both *EphA2^+/+^* and *EphA2^-/-^* mice, lens aspect ratio decreases with age. **(D)***EphA2^-/-^* nucleus volumes are smaller than controls at all age groups. In both *EphA2^+/+^* and *EphA2^-/-^* lenses, nucleus volumes increase with age. *P<0.05; **P<0.01; ***P<0.001; ****P<0.0001.

We measured biomechanical properties and morphometrics of EphA2 and ephrin-A5 control and respective KO lenses from 4- and 8-month-old mice. We compared data from these older adult mice with data from our previous study on 2-month-old control and KO mice (5, 11). Prior work has shown anterior cataracts, cortical haziness, and ring cataracts are observed in lenses from C57BL/6J *WT* mice older than 10–12 months (29). Due to these age-related defects, we did not study mice older than 8 months of age for this work. For these measurements, we imaged eyes before microdissection and lenses before compression, during compression using our simple coverslip compression method, and post-compression to calculate resilience or recovery after load removal. We also isolated the lens nucleus for imaging after compression experiments. Whole eye images did not reveal obvious defects in KO eyes (**Figures 1**, **2**). In side-view images of control and KO lenses, we observed that control and KO lenses were compressed similarly under 1 coverslip, 5 coverslips, and 10 coverslips (**Figures 1**, **2**). We noticed that *EphA2^-/-^* lens nuclei were smaller than control lens nuclei at all ages (**Figure 1**, red arrowheads). We measured and calculated eye volume, lens volume, lens aspect ratio (axial/equatorial diameter), and nucleus volume. In *EphA2^+/+^* mice, eye volume increases with age, while *EphA2^-/-^* eye volume increases between 2 and 4 months of age and does not change significantly between 4 and 8 months of age (**Figure 3A**). *EphA2^-/-^* mice have smaller eye volumes when compared to controls at all ages. Lens volumes increased with age in control mice, as expected. But *EphA2^-/-^* lenses did not increase in size between 2 and 4 months of age, and KO lenses were smaller than controls at 4 and 8 months of age (**Figure 3B**). Lens aspect ratio decreased, and lenses became more spherical with age in control and KO mice. The shape of *EphA2^-/-^* lenses was closer to sphericity than those of control lenses at all ages (**Figure 3C**). The volume of the lens nucleus increases with age in both control and *EphA2^-/-^* lenses; however, *EphA2^-/-^* mice had smaller lens nuclei at all ages (**Figure 3D**). Changes in lens volume and nuclear volume with age in control mice agree with our previous lens morphometrics study of C57BL/6J *WT* mice (10, 29). The smaller lens size for 4- and 8-month-old *EphA2^-/-^* mice suggests there is an adult-onset growth defect, and these data suggest that EphA2 influences lens shape and is required for normal eye, lens, and nuclear size.

**Figure 4:**
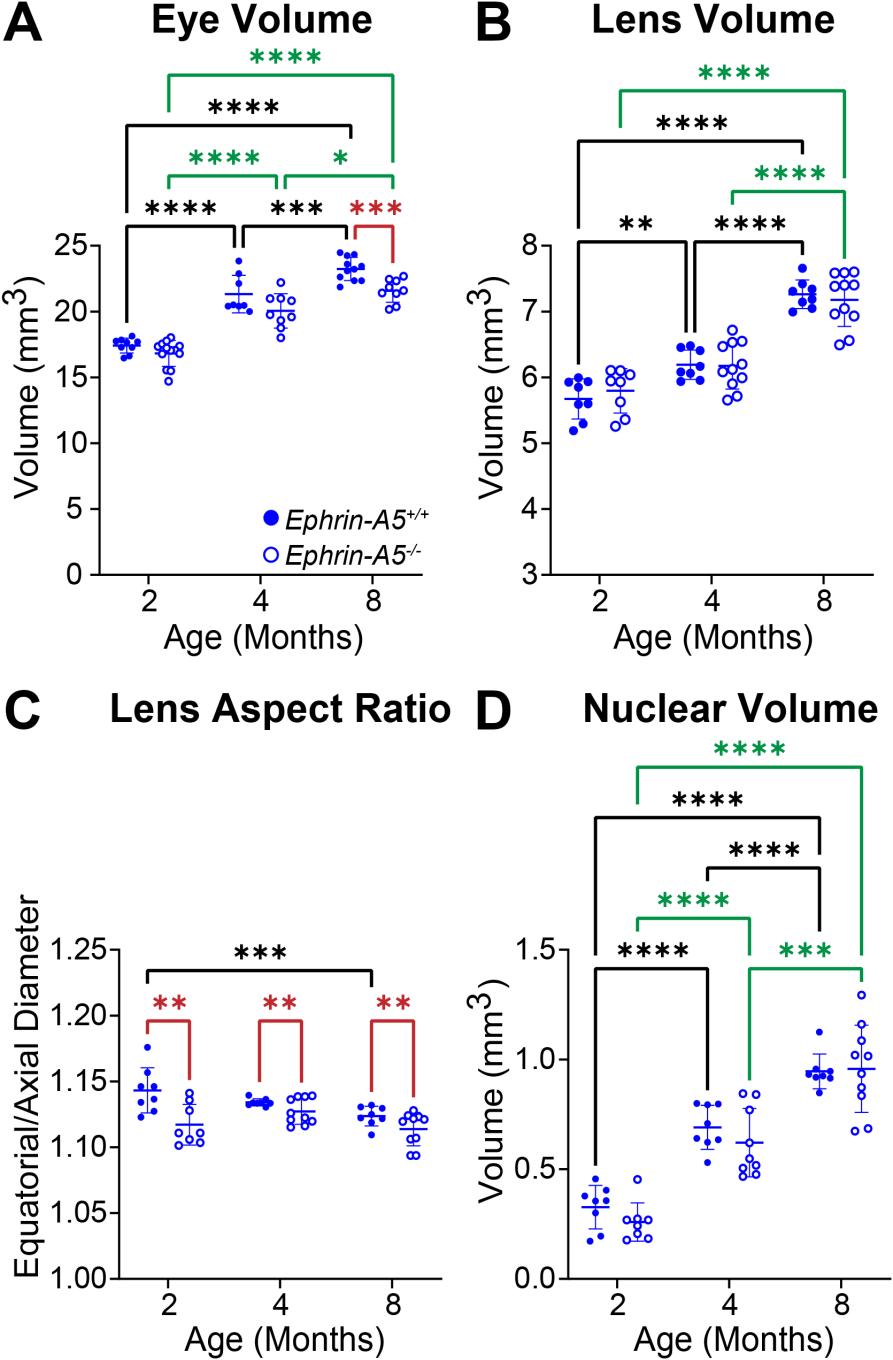
Morphometric measurements of eyes, lenses, and lens nuclei from 2-, 4-, and 8-month-old *ephrin-A5^+/+^* and *ephrin-A5^-/-^* mice. Data from the 2-month-old mice are replotted from our previous work (11). Data on the graphs reflect the mean ± SD from at least 8 lenses per age and genotype. **(A)** Eye volume is smaller in 8-month-old e*phrin-A5^-/-^* mice compared to controls. In *ephrin-A5^+/+^* and *ephrin-A5^-/-^* mice, eye volume increases with age. **(B)** Lenses from control mice increase in volume with age, but *ephrin-A5^-/-^* lenses do not significantly increase in size between 2- and 4- months of age. There are no significant differences between control and KO lenses in any age group. **(C)***Ephrin-A5^-/-^* lenses are more spherical than control lenses in all age groups. There is no difference in lens aspect ratio with age except between 2- and 8-month-old control mice, where there is a decrease in this parameter. **(D)** Nucleus volumes increase with age in both control and *ephrin-A5^-/-^* mice, and there are no differences between control and KO lens nuclei. *P<0.05; **P<0.01; ***P<0.001; ****P<0.0001.

In comparison, control and *ephrin-A5^-/-^* eyes increase in volume with age, and *ephrin-A5^-/-^* mice have slightly smaller eye volumes only at 8 months of age (**Figure 4A**). Lens volume increase with age in control *ephrin-A5^+/+^* mice (**Figure 4B**). *Ephrin-A5^-/-^* lens volumes were comparable between 2- and 4-months of age, but KO lenses increased in volume between 4- and 8-months of age (**Figure 4B**). There were no significant changes in lens volume in *ephrin-A5^-/-^* mice when compared to controls (**Figures 4B**). In *ephrin-A5^+/+^* control mice, there is a slight decrease in lens aspect ratio only between 2- and 8-month-old animals (**Figure 4C**). While lens shape does not change significantly with age in *ephrin-A5^-/-^* mice, *ephrin-A5^-/-^* lenses are more spherical than control lenses at all ages (**Figure 4C**). Nucleus volume increases with age in both control and *ephrin-A5^-/-^* mice, and there was no significant difference between KO and control lens nuclei at any age (**Figure 4D**). These data showed that disruption of either EphA2 or ephrin-A5 led to more spherical lenses and affected normal lens growth between 2 and 4 months of age.

### 3.2 Differences in lens biomechanical properties were detected in 8-month-old *EphA2^-/-^* mice

**Figure 5:**
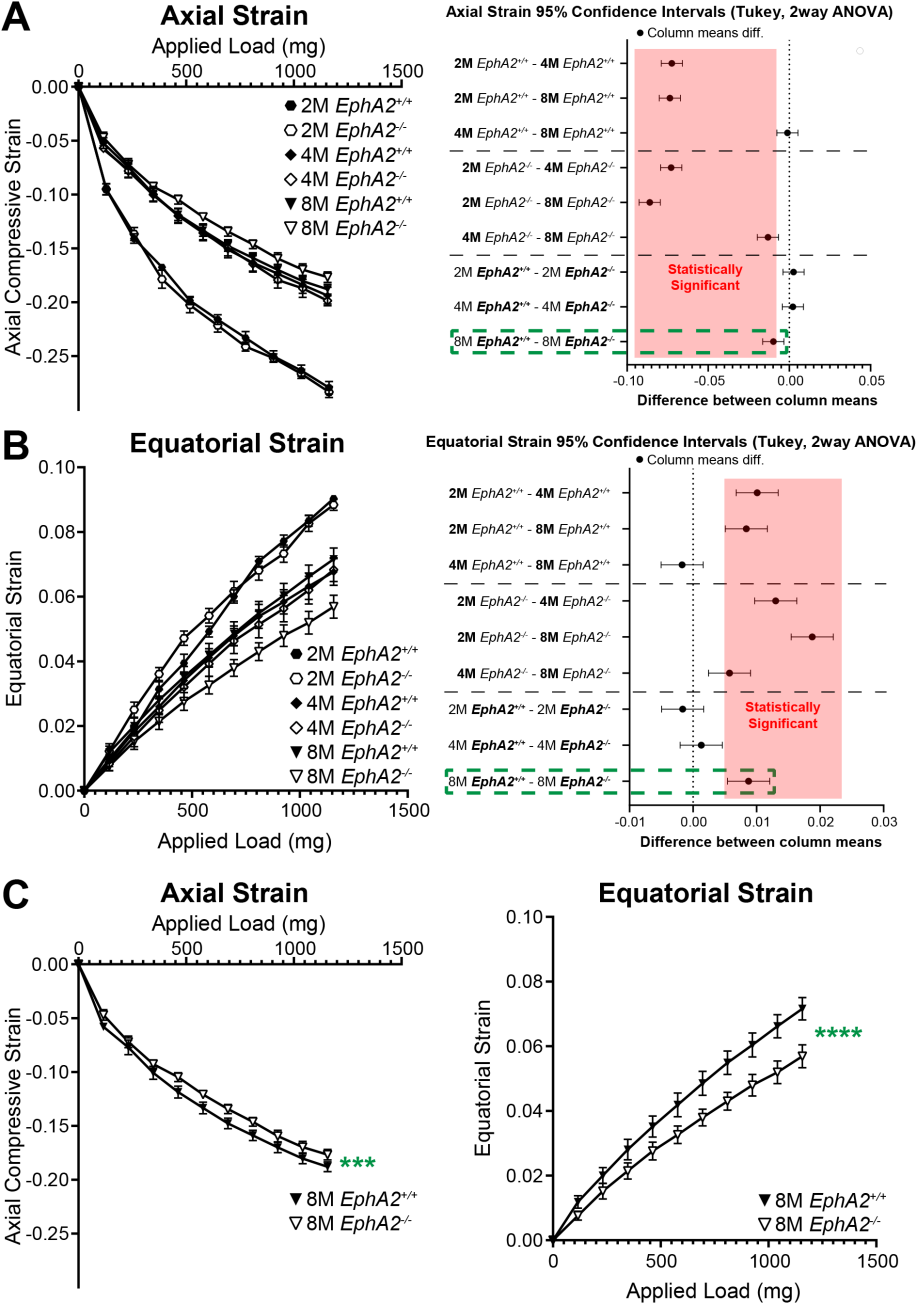
Axial and equatorial strain of lenses under compression from *EphA2^+/+^* and *EphA2^-/-^* at 2–8 months of age. Data from the 2-month-old mice are replotted from our previous work (11). Plots reflect mean ± SD from at least 8 lenses per age and genotype. To the right of each strain plot is a graph showing the 95% confidence interval for significant differences between genotypes or age groups. Any comparisons not crossing the dotted line are statistically significant (*p*<0.05) (light red box). Compression testing using sequential application of coverslips showed a decrease in axial **(A)** and equatorial strain **(B)** with age, indicating that lenses from older mice are stiffer. There was stiffening of the lens between 2 and 4 months of age, but not between 4 and 8 months of age, in control mice. Between *EphA2^-/-^* and control lenses, there were no significant differences except at 8 months of age (dotted green boxes). **(C)** Eight-month-old *EphA2^-/-^* lenses are stiffer than the respective control group when we compare axial and equatorial strains. ***P<0.001; ****P<0.0001.

**Figure 6:**
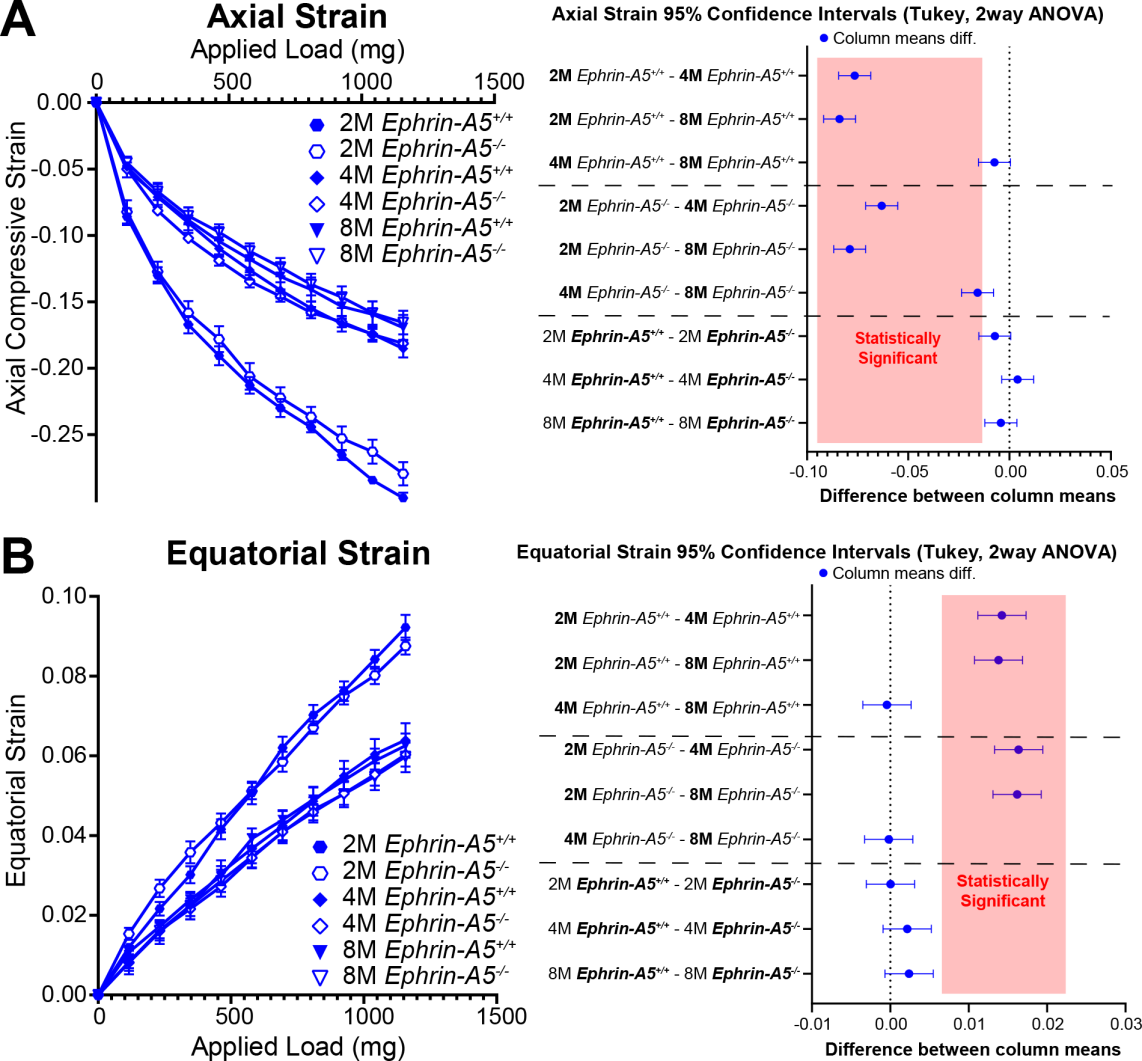
Axial and equatorial strain of lenses under compression from *ephrin-A5^+/+^* and *ephrin-A5^-/-^* at 2–8 months of age. Data from the 2-month-old mice are replotted from our previous work (11). Plots reflect mean ± SD from at least 8 lenses per age and genotype. To the right of each strain plot is a graph showing the 95% confidence interval for significant differences between genotypes or age groups. Any comparisons not crossing the dotted line are statistically significant (*p*<0.05) (light red box). Compression testing using sequential application of coverslips showed a decrease in axial **(A)** and equatorial strain **(B)** with age, indicating that lenses from older mice are stiffer. There was stiffening of the lens between 2 and 4 months of age in control and *ephrin-A5^-/-^* lenses. Axial and equatorial strains were not different between control and KO lenses from the same age group.

Our previous data in 2-month-old mice revealed no changes in lens stiffness due to disruption of EphA2 or ephrin-A5, but resilience increased significantly in both KO lenses compared to respective controls (5). We further explored whether loss of Eph-ephrin signaling affects lens stiffness and resilience with age. With sequential application of coverslips to increase the applied load, both axial and equatorial strain increased as expected (**Figures 5**, **6**). Lenses from older mice show reduced strain at high loads because mouse lenses stiffen with age (29). There was a non-linear relationship between strain and applied load due to tissue mechanics factors including creep and viscoelasticity (10). Axial strains are negative due to the compressive load that decreases the axial diameter of the lens (**Figures 5A**, **6A**), while equatorial strains are positive due to the expansive force that increases the equatorial diameter of the lens during compression (**Figures 5B** and **6B**). Using two-way Anova analysis, the 95% confidence intervals for strain difference are plotted to the right of each strain plot (**Figures 5A, B**, **6A, B**). The upper 1/3 of the graphs are changes due to age in control lenses, the middle 1/3 of the graphs display changes due to age in KO lenses, and the lower 1/3 of the graphs shows changes between control and KO lenses for each age group. Control *EphA2^+/+^* and *ephrin-A5^+/+^* lenses increase in stiffness between 2 and 4 months of age and do not stiffen significantly between 4 and 8 months of age. In *EphA2^-/-^* mice, lenses increase in stiffness between 2, 4, and 8 months of age. *Ephrin-A5^-/-^* lenses appeared to stiffen with age when examining the axial strain, but there was no change in equatorial strain of *ephrin-A5^-/-^* lenses between 4 and 8 months of age. This discrepancy is likely due to equatorial expansion changes occurring at a smaller scale than axial compressive strain with applied loads. *Ephrin-A5^-/-^* lenses were stiffer in 8- vs. 4-month-old mice at least in the axis of the applied load. There were no differences between control and KO groups except for 8-month-old *EphA2^-/-^* lenses that displayed increased stiffness with biomechanical testing compared to the respective control group (**Figures 5A, B**, green dashed boxes and **Figure 5C**).

**Figure 7:**
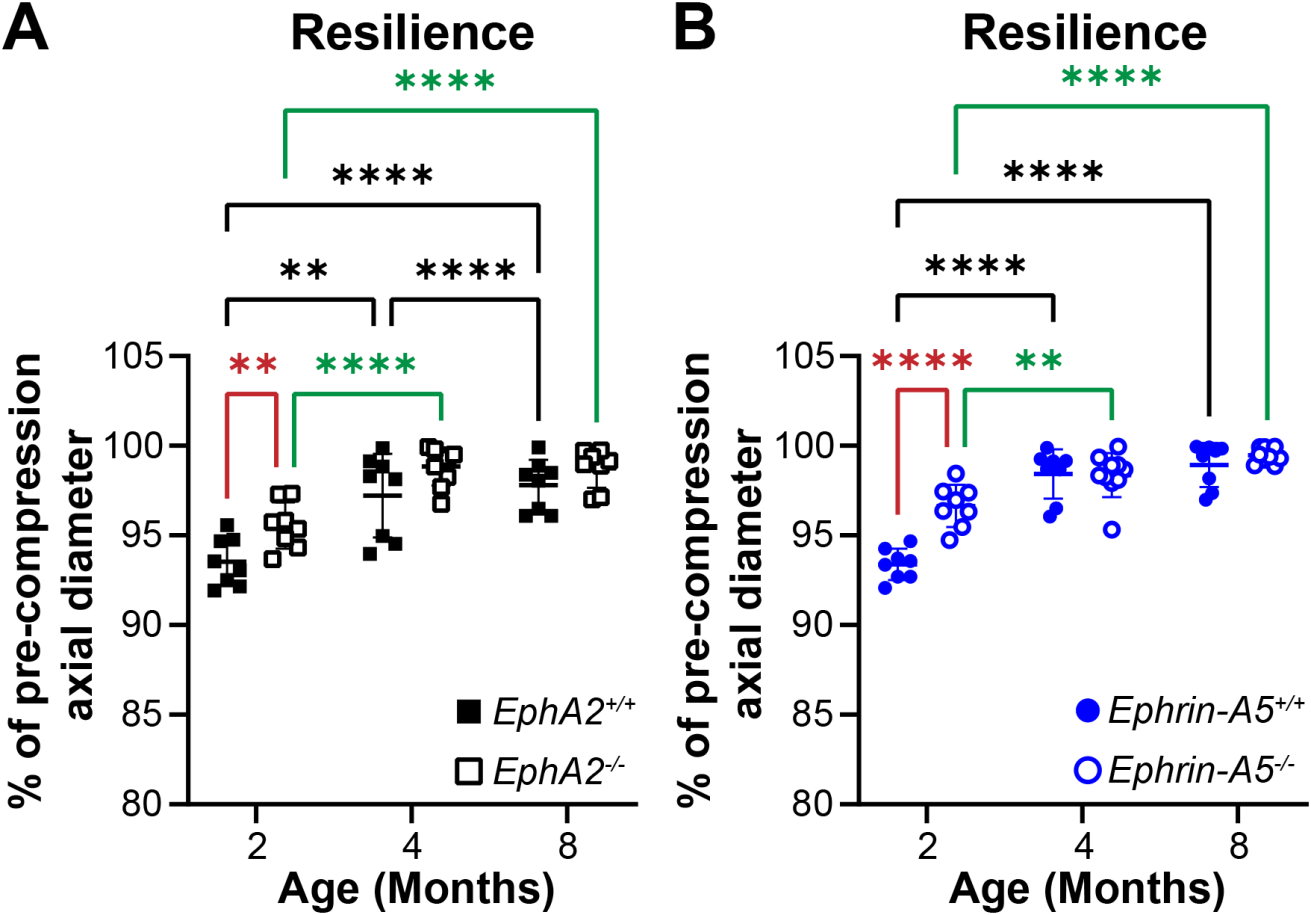
Resilience of *EphA2^+/+^*, *EphA2^-/-^*, *ephrin-A5^+/+^*, and *ephrin-A5^-/-^* lenses from mice between ages 2–8 months of age. Data from the 2-month-old mice are replotted from our previous work (11). Plots reflect mean ± SD from at least 8 lenses per age and genotype. Resilience was calculated as the percent ratio of pre-compressed over post-compressed axial diameter. **(A, B)** In 2-month-old *EphA2^-/-^* and *ephrin-A5^-/-^* mice, lenses had increased resilience compared to the respective control group. However, in older mice, there is no obvious difference in resilience between respective control and KO lenses. Resilience increased between 2 and 4 months of age for all genotypes, and resilience also increased between 4 and 8 months of age in *EphA2^+/+^* lenses. **P<0.01; ****P<0.0001.

In addition to strain measurements, we also determined the resilience of the lens using the ratio between pre- and post-compression axial diameter after load removal. We previously showed that *EphA2^-/-^* and *ephrin-A5^-/-^* lenses displayed increased resilience after load removal in 2-month-old mice (5). This difference is absent between older control and KO mice (**Figure 7**). Resilience generally increases with age in control and KO lenses.

### 3.3 GRIN and max refractive index were decreased in *EphA2^-/-^* lenses

**Figure 8:**
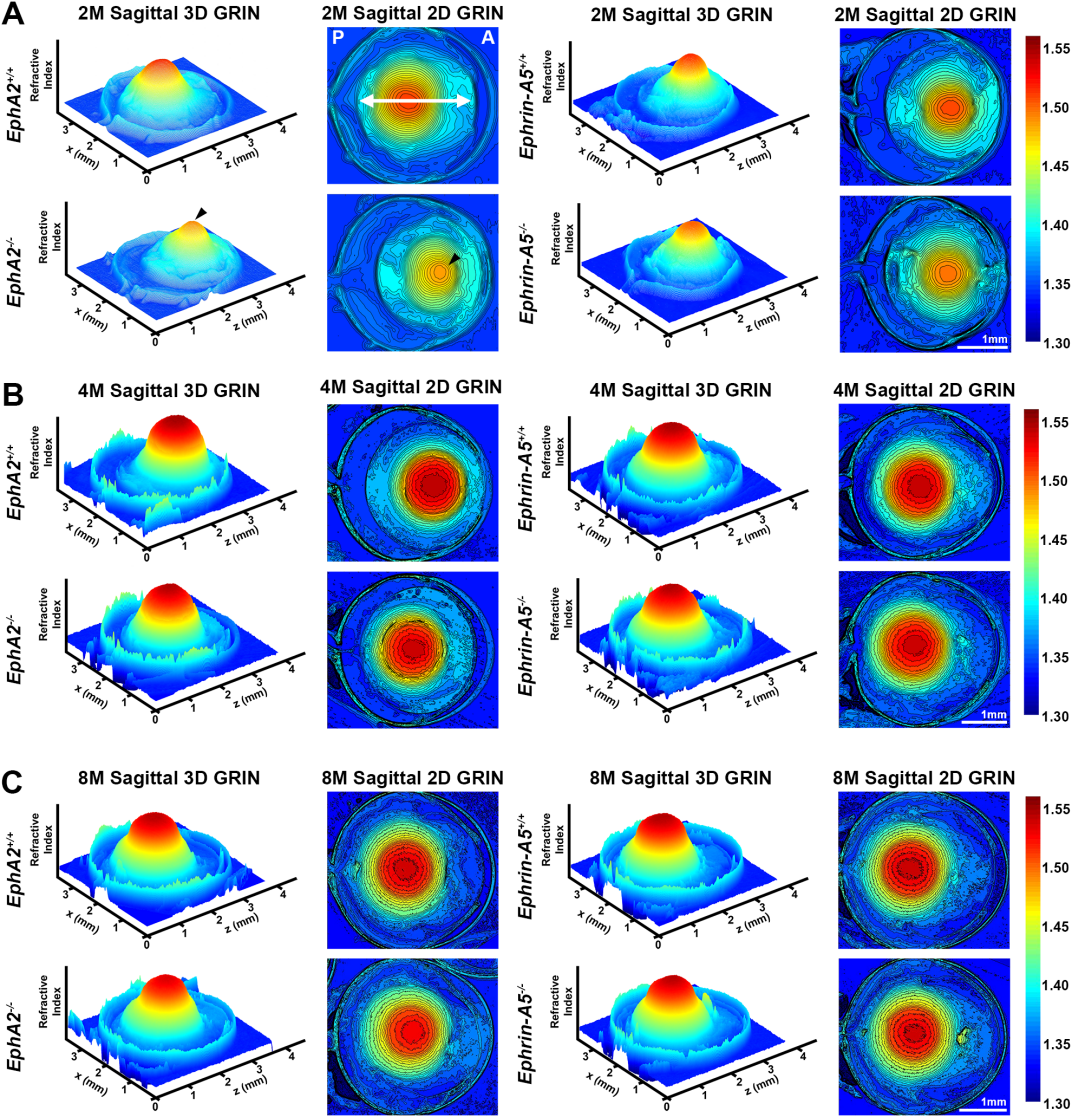
Representative GRIN 3D mesh and 2D contour plots of whole eyes from *EphA2^-/-^* and *ephrin-A5^-/-^* mice and their respective controls. 2D contour plots are shown through the mid-sagittal plane (anterior-posterior plane through the visual axis). Data from the 2-month-old mice are replotted from our previous work (11). A rainbow gradient of colors reflects that magnitude of refractive index from low refractive index in dark blue (1.30) to high refractive index in dark red (1.55). **(A)** The anterior (A) and posterior of the eye (P) are marked on the sagittal view of the 2-month-old control eye. The double arrow indicates the approximate location of the lens. **(A)** Lenses from 2-month-old *EphA2^-/-^* mice have obviously decreased GRIN when compared to control 3D and 2D plots (arrowheads). The central region of *EphA2^-/-^* lenses is orange, rather than deep red as seen in controls, indicating a lower refractive index in *EphA2^-/-^* lenses. There are no obvious differences between 3D and 2D GRIN plots from 4-month-old and 8-month-old control and *EphA2^-/-^* mice. **(B, C)** In comparison, 2-, 4-, and 8-month-old control and *ephrin-A5^-/-^* mice have comparable 3D and 2D GRIN plots **(A–C)**. Scale bars, 1mm.

**Figure 9:**
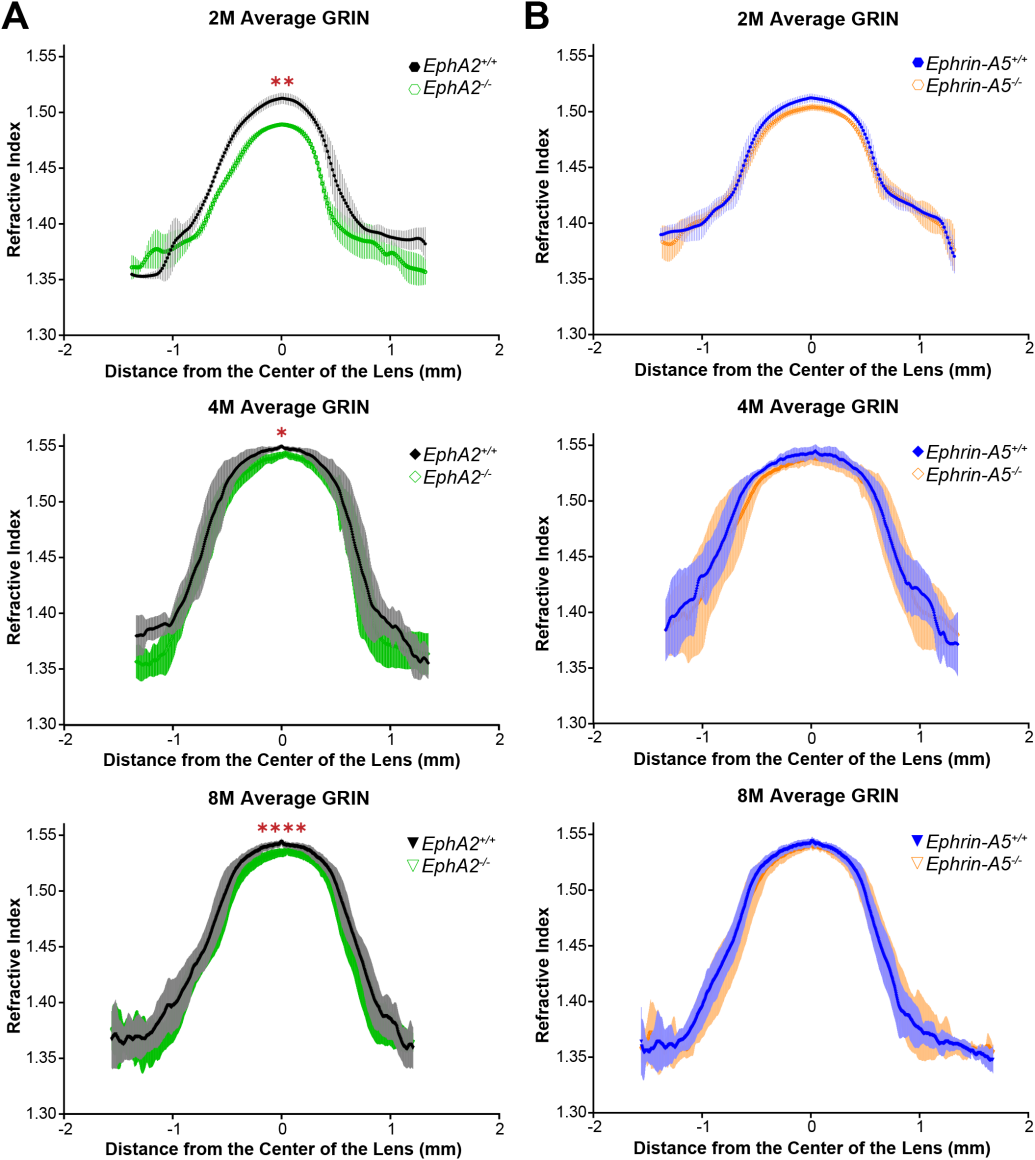
GRIN profile from lenses along the visual axis plotted as the mean ± SD. Data is from at least 6 lenses per age and genotype. Data from the 2-month-old mice are replotted from our previous work (11). **(A)** The average and standard deviation of the GRIN profile are plotted for *EphA2^+/+^* and *EphA2^-/-^* lenses from 2-, 4-, and 8-month-old mice. *EphA2^-/-^* lenses have decreased GRIN in all three age groups. **(B)** There are no significant differences in GRIN between control and *ephrin-A5^-/-^* mice in any age group. *P<0.05; **P<0.01; ****P<0.0001.

**Figure 10:**
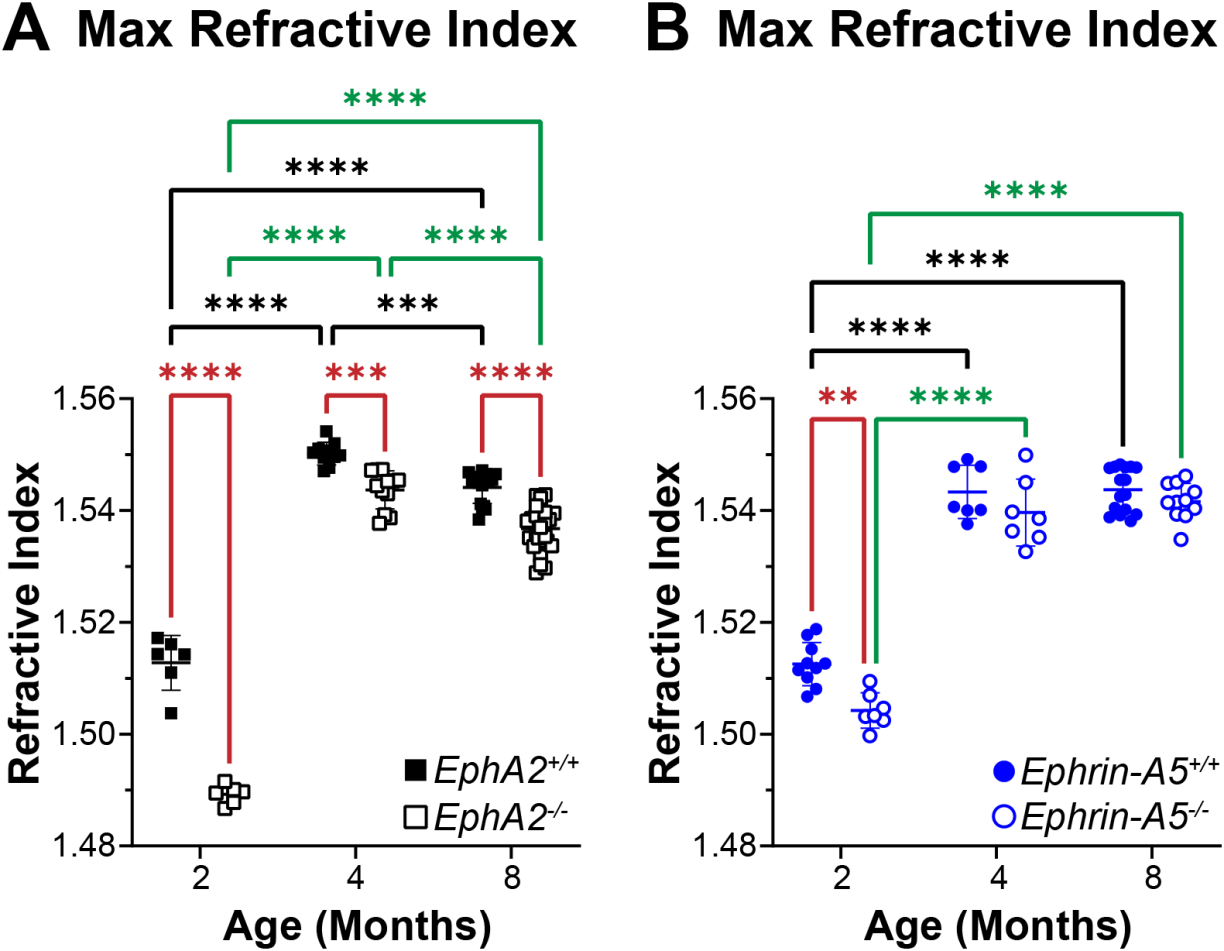
Max refractive index plots from lenses of 2-, 4-, and 8-month-old, control, *EphA2^-/-^*, and *ephrin-A5^-/-^* mice. Data from the 2-month-old mice are replotted from our previous work (11). Plots represent the mean ± SD from at least 6 lenses per age and genotype. **(A)***EphA2^-/-^* lenses had significantly decreased max refractive index at the center of the lens when compared to controls. Max refractive index increases between 2 and 4 months of age but decreases between 4and 8 months of age in control and *EphA2^-/-^* lenses. **(B)** While there is a slight decrease in max refractive index in lenses from 2-month-old *ephrin-A5^-/-^* mice, max refractive index is comparable between lenses from 4- and 8-month-old control and *ephrin-A5^-/-^* mice. Max refractive index increases between 2 and 4 months age and is not different between 4 and 8 months of age in control and *ephrin-A5^-/-^* lenses. **P<0.01; ***P<0.001.****P<0.0001.

In addition to biomechanical properties, GRIN contributes to fine focusing of light onto the retina and is correlated with the size of the lens nucleus (11, 29). We previously showed that the smaller lens nuclei in 2-month-old *EphA2^-/-^* mice resulted in decreased GRIN and max refractive index in those KO lenses and that loss of ephrin-A5 also led to slightly decreased max refractive index in 2-month-old mice (11). Therefore, we measured GRIN in lenses from 4-month-old and 8-month-old control, *EphA2^-/-^*, and *ephrin-A5^-/-^* mice and generated 2D and 3D contour plots in the sagittal plane (**Figure 8**) and plotted the GRIN across the visual axis (**Figure 9**). The 2D and 3D contour plots are a heat map of the refractive index with high refractive index in red and low refractive index in blue. While there was an obvious decrease in refractive index across eyes from 2-month-old *EphA2^-/-^* mice (**Figure 8A**, left, arrowheads), in *EphA2^-/-^* and *ephrin-A5^-/-^* eyes from 4-month-old and 8-month-old mice, the GRIN contour plots appeared similar to respective control eyes (**Figures 8B, C**). When we plotted the average and standard deviations of the GRIN across the lens in the visual axis, we observed a slight but significant decrease in the GRIN in *EphA2^-/-^* lenses from all ages (**Figure 9A**). There were no observed changes in GRIN in *ephrin-A5^-/-^* mice (**Figure 9B**). When we compared the max refractive index at the center of the lens, we observed an increase in max refractive index between *EphA2^+/+^* and *EphA2^-/-^* lenses from 2- and 4-month-old mice, but the max refractive index decreased slightly between 4 and 8 months of age (**Figure 10A**). *EphA2^-/-^* lenses had decreased max refractive index compared to controls at all ages (**Figure 10A**). In *ephrin-A5^+/+^* and *ephrin-A5^-/-^* lenses, max refractive index increased between 2 and 4 months of age, and there were no changes between 4 and 8 months of age (**Figure 10B**). While the max refractive index slightly decreased in lenses from 2-month-old *ephrin-A5^-/-^* mice, the max refractive index was not significantly different between control and *ephrin-A5^-/-^* lenses from 4-month-old and 8-month-old mice (**Figure 10B**). These data suggest that EphA2 likely influences the GRIN by determining the properties of the lens nucleus.

## 4 Discussion

Our data shows that loss of EphA2 resulted in smaller eye and lens size, a more spherical lens, and a smaller lens nucleus, and these changes were observed across all three age groups (**Table 1**). In contrast, ephrin-A5 deletion led to more spherical lens in all age groups. Differences in lens resilience between control and KO mice were only apparent at 2 months of age. Despite changes in lens morphometrics between respective control and KO groups, there were no differences in lens stiffness for all comparisons except between lenses from 8-month-old control and *EphA2^-/-^* mice where the deletion of EphA2 resulted in increased lens stiffness. These data show that decreased lens and nucleus size do not lead to softer lenses and contradicts previous hypotheses that increased lens and/or nucleus size are the cause for age-related increases in lens stiffness (8, 9, 24–28).

**Table 1:** Comparison of lens biomechanics, morphometrics, and GRIN between control and KO lenses from 2-, 4-, and 8-month-old mice.

Comparison made	Age	Lens stiffness	Lens resilience	Eye volume	Lens volume	Lens aspect ratio	Nucleus volume	GRIN	Max refractive index
*EphA2^-/-^* compared to *EphA2^+/+^*	2M	~	↑	↓	~	↓	↓	↓	↓
	4M	~	~	↓	↓	↓	↓	↓	↓
	8M	↑	~	↓	↓	↓	↓	↓	↓
*Ephrin-A5^-/-^* compared to *Ephrin-A5^+/+^*	2M	~	↑	~	~	↓	~	~	↓
	4M	~	~	~	~	↓	~	~	~
	8M	~	~	↓	~	↓	~	~	~

Data for 2-month-old mice were collected previously (5, 11). Statistically significant increases are indicated by a green up arrow while decreases are indicated by a red down arrow. A tilde symbol is displayed when there is no change between control and KO lenses.

Beyond lens and nucleus size, other factors, including the lens capsule, cytoskeletal networks, plasma membrane lipids, protein aggregation, and hydrostatic pressure, can influence age-related changes in lens biomechanics [reviewed in (63)]. During fiber cell maturation, the cells form complex interdigitations with each other. It has been shown that loss of these interdigitations results in a softer and more easily compressed lens (54, 64). In young adult mice, *EphA2^-/-^* lens fibers have abnormal interdigitations (11), and further studies using electron microscopy (11, 29) or single fiber cell staining (64–66) will be needed to determine whether there are additional changes in fiber cell packing and shape with age. Lens fiber cell shape and patterning relies on the F-actin network (67), and changes in the F-actin network composition or disruption of the network through pharmacological treatment can also affect lens stiffness (65, 68). EphA2 is known to regulate the actin cytoskeleton in equatorial lens epithelial cells to determine cell shape and organization (48). The effects on the F-actin cytoskeleton in *EphA2^-/-^* lens fiber cells remain an area of active research.

When we compared the morphometric and biomechanics data across different age groups within the same genotype and with data from our previous works (5, 10, 11, 29), we found that while there was general agreement, there are some differences between the controls in our studies and C57BL/6J *WT* mice (**Table 2**). While lens stiffness continued to increase with age in *WT* mice (10, 29), interestingly, lens stiffness does not increase in control (*EphA2^+/+^* and *ephrin-A5^+/+^*) and *ephrin-A5^-/-^* mice between 4 and 8 months of age (**Figures 5**, **6**). Lenses from *EphA2^-/-^* mice increase in stiffness between 4 and 8 months of age, and this is likely driving the increased lens stiffness compared to controls at 8 months of age. Further investigation is required to determine the lack of age-related stiffness changes in our control lens from 4 to 8 months of age. Data from C57BL/6J *WT* mice did not identify significant changes in lens resilience between 2, 4, and 8 months of age (29). We observe an increase in lens resilience in the control and KO mice in this study between 2 and 4 months of age, with no additional increase in resilience between 4 and 8 months of age except in *EphA2^+/+^* lenses. While we have backcrossed our mice frequently and for many generations with C57BL/6J *WT* mice, it is not clear why data from our control mice does not match the resilience data from C57BL/6J *WT* mice. It is possible that mouse genetic background influences lens resilience. Our previous work also suggests that the patterning and organization of the Y-suture in mouse lenses regulates lens resilience, and *EphA2^-/-^* and *ephrin-A5^-/-^* lenses have highly branched and mispatterned sutures while control lenses have well-defined Y-shaped sutures (5). Changes of the Y-suture with age in control and KO lenses require additional future studies.

**Table 2:** Changes in lens biomechanics, morphometrics, and GRIN with age.

Genotype	Age range	Lens stiffness	Lens resilience	Eye volume	Lens volume	Lens aspect ratio	Nucleus volume	Age range	GRIN	Max refractive index
Wild-type (C57BL/6J) (10)	2M-4M	↑	N/A	N/A	↑	~	↑		N/A	N/A
	2M-8M	↑	N/A	N/A	↑	~	↑		N/A	N/A
	4M-8M	↑	N/A	N/A	↑	~	↑		N/A	N/A
Wild-type (C57BL/6J Albino) (29)	2M-4M	↑	~	N/A	↑	↑	↑	6W-3M	↑	↑
	2M-8M	↑	~	N/A	↑	↑	↑	6W-8M	↑	↑
	4M-8M	↑	~	N/A	↑	~	↑	3M-8M	↑	↑
*EphA2^+/+^* [2M data (5, 11)]	2M-4M	↑	↑	↑	↑	↓	↑		↑	↑
	2M-8M	↑	↑	↑	↑	↓	↑		↑	↑
	4M-8M	~	↑	↑	↑	↓	↑		~	↓
*Ephrin-A5^+/+^* [2M data (5, 11)]	2M-4M	↑	↑	↑	↑	~	↑		↑	↑
	2M-8M	↑	↑	↑	↑	↓	↑		↑	↑
	4M-8M	~	~	↑	↑	~	↑		~	~
*EphA2^-/-^* [2M data (5, 11)]	2M-4M	↑	↑	↑	~	↓	↑		↑	↑
	2M-8M	↑	↑	↑	↑	↓	↑		↑	↑
	4M-8M	↑	~	~	↑	↓	↑		~	↓
*Ephrin-A5^-/-^* [2M data (5, 11)]	2M-4M	↑	↑	↑	~	~	↑		↑	↑
	2M-8M	↑	↑	↑	↑	~	↑		↑	↑
	4M-8M	↑	~	↑	↑	~	↑		~	~

Comparison of data collected in this study and previous works (5, 10, 11, 29). Statistically significant increases with age are indicated by a green up arrow while decreases with age are indicated by a red down arrow. A tilde symbol is displayed when there is no change between the age groups. Some prior studies did not measure every parameter in the table, and those are marked as N/A.

Our measurements of eye volume reveal an increase between 2- and 4-month-old *ephrin-A5^+/+^*, *EphA2*^-/-^, and *ephrin-A5^-/-^* mice (**Table 2**). Eye size does not further increase between 4 and 8 months of age for these genotypes. However, in *EphA2^+/+^* mice, there was an increase in eye volume between all three age groups. It is unclear why these control mice differ from the other three genotypes. Lens volumes increased with age in control mice, in agreement with previous data (10, 29). There was no change in lens volume for *EphA2^-/-^* and *ephrin-A5^-/-^* mice between 2 and 4 months of age. The lack of significant lens growth between 2- and 4-month-old *EphA2^-/-^* and *ephrin-A5^-/-^* mice suggests that Eph-ephrin signaling is required for normal adult lens growth. Though the exact mechanism remains to be explored, it is possible that mispatterned and abnormal sutures of *EphA2^-/-^* and *ephrin-A5^-/-^* lens fiber cells (47) can alter the addition of new layers of secondary fibers that must be overlaid onto previous generations of fiber cells. Alternatively, proliferation rate and/or cell death rate in equatorial KO epithelial cells may also need to be studied to understand whether the growth defect originates from a germinative zone epithelial cell defect. Previous work has shown that the growth of the eye is determined by expansion of the vitreous, and accumulation of the vitreous increases in response to factors from the lens, though little is known about the mechanisms the lens utilizes to promote this action (69). Additionally, data from human and animal studies reveal that the loss or absence of the lens results in a small eye (70). Our data shows loss of EphA2 results in a smaller lens volume at 4 and 8 months of age (**Table 1**). At the same time, eye volume in *EphA2^-/-^* mice does not increase from 4 to 8 months (**Table 2**). While we did not include *ephrin-A5^-/-^* mice with microphthalmia in this study, we observed *ephrin-A5^-/-^* mice with smaller lens volumes also had smaller eye volumes (data not shown). This further supports the idea that lens growth influences eye growth and size. It is worth noting that 2-month-old *EphA2^-/-^* mice and 8-month-old *ephrin-A5^-/-^* mice had decreased eye volume compared to their age-matched controls without any change in lens volume. It is possible that disruption of Eph-ephrin signaling leads to changes in other ocular tissues that affects whole eye growth.

We detect decreased lens aspect ratio in *EphA2^+/+^* and *EphA2^-/-^* lenses with age, and while there is a general trend for lenses to become more spherical in shape with age in other genotypes, lens aspect ratio does not change with age in one cohort of *WT* mice (10) and in *ephrin-A5^-/-^* lenses. It is unclear why the lens aspect ratio data from the two *WT* mice studies differ, but those mice were not in the exact same genetic background (10, 29). For the *ephrin-A5^-/-^* mice, it is possible that the lens shape is already quite spherical at 2 months of age, and thus, with age, the initial shape change is maintained when more fiber cells are added and overlaid onto previous generations of cells. Alternatively, in this ephrin-A5 mouse line, there are only subtle changes in *ephrin-A5^+/+^* lens shape between 2 and 8 months, and the KO lenses may grow similarly to the respective control lenses that do not change much in shape with age. Lens shape is determined by fiber cell curvature and lens suture patterning. It has been observed in developing rodent lenses that fiber cells begin in a concave curvature, then straighten, and convert to a convex curvature, which gives rise to the whole lens ellipsoid shape (71). When Sfrp2, a Wnt signaling antagonist, is overexpressed in fiber cells, there is reduced cell elongation, irregular fiber shapes that fail to pack properly, and an absence in normal convex curvature of lens fibers (72). These fiber cell defects result in abnormal lens shape in Sfrp2 mutant mice. In lenses from different animal species, the type of suture (Y-shaped, umbilical, line, or branched) is hypothesized to be correlated with lens shape (71). Our previous data (11) and this work reveal Eph-ephrin signaling also affects lens shape. Control lenses have a distinct Y-shaped pattern with rare occurrences of branching whereas EphA2 or ephrin-A5 KO lenses have suture patterns with many branches that are misaligned between shells of fiber cells (5). The altered suture pattern in EphA2 or ephrin-A5 KO lenses is linked to a more spherical tissue shape. Interestingly, suture patterns of EphA2 and ephrin-A5 KO lenses more closely resemble branched sutures in human lenses (73, 74) that become more spherical with age.

In general, lens and nucleus volumes are lower in *EphA2^-/-^* mice when compared to controls, and there are no differences between ephrin-A5 control and KO lenses. In 2-month-old *EphA2^-/-^* mice and in *ephrin-A5^-/-^* mice, lens volume is not significantly changed compared to age-matched controls, but the shape of KO lenses was more spherical. These data suggest that despite the shape change, there is conservation of lens volume, and the shape of the lens does not necessarily affect the size of the lens. Interestingly, *EphA2^-/-^* lenses with lower volume and smaller nuclei were stiffer than control lenses at 8 months of age. Our morphometrics and biomechanics data show lens and nucleus volumes are not directly correlated with stiffness changes, in agreement with data from previous works (5, 11, 13, 65). To determine whether lens growth and nucleus growth are coupled in adult lenses, we have calculated nucleus fraction by dividing nucleus volume by lens volume. The nucleus fraction in control *EphA2^+/+^* lenses is larger than *EphA2^-/-^* lenses at all ages (data not shown). This result suggests that while both control and *EphA2^-/-^* lens and nucleus volumes increase with age, *EphA2^-/-^* nuclei growth does not catch up to control lens nuclei during adult lens growth, and the smaller KO nucleus does not occupy the same proportional volume in the smaller KO lens. Using our coverslip method, we are unable to assess the stiffness of the lens nucleus, and we are working on alternate methods to determine nucleus stiffness to determine how nucleus stiffness affects whole lens stiffness. Our previous work showed that nuclei of *EphA2^-/-^* lenses from 2-month-old mice can be deformed by gloved fingertips compared to very hard control lens nuclei (11), and the softer *EphA2^-/-^* lens nucleus did not affect lens stiffness (5). Thus, it seems unlikely that lens nucleus stiffness directly contributes to whole lens stiffness, at least in rodent lenses.

Differences in GRIN are apparent in the EphA2 strain with the *EphA2^-/-^* lenses having lower average GRIN and lower max refractive index. Average GRIN is not affected by the loss of ephrin-A5. While max refractive index decreases in *ephrin-A5^-/-^* lenses at 2 months of age, there were no differences between KO and control max refractive index in lenses from older mice. Since nucleus size is correlated with the establishment of high refractive index in the lens (11, 29), it is not surprising that loss of EphA2 leads to decreased average GRIN, and our data further supports the influence of nucleus size on GRIN. Previous work in *WT* mice show that average GRIN and max refractive index increases until 6 months of age, then plateaus (29). In the EphA2 strain, average GRIN and max refractive index increases between 2 to 4 months of age. Surprisingly, the average GRIN plateaus and max refractive index decreases between 4 to 8 months of age in *EphA2^+/+^* and *EphA2^-/-^* mice. In the ephrin-A5 strain, average GRIN and maximum refractive index increases between 2 to 4 months of age, then plateaus. It is not clear why max refractive index decreases in EphA2 strain mice between 4 and 8 months, and additional experiments to explore protein content in lens nuclei [reviewed in (12)] may be required to understand the changes in max refractive index with age. The differences between our *EphA2^+/+^* and *ephrin-A5^+/+^* controls and prior *WT* data for various morphometric, biomechanical, and refractive index measurements demonstrate the need to use littermate controls rather than just a *WT* mouse strain as a comparison since the differences in between control and *WT* phenotypes can lead to misinterpretation of data.

While EphA2 and ephrin-A5 are a receptor-ligand pair in other tissues and cells (75–77), our previous work (49) and the data from this study suggest that these two proteins do not form an exclusive binding pair. EphA2 KO and ephrin-A5 KO lenses share some similarities in changes in lens shape that are likely the result of changes in the lens suture, where our prior results suggest a possible interaction between EphA2 and ephrin-A5 (5). However, in most other morphometric measurements, *EphA2^-/-^* lenses differ from *ephrin-A5^-/-^* lenses, providing further evidence that EphA2 and ephrin-A5 are not an exclusive receptor-ligand pair in the lens.

In summary, this work shows that decreased lens size and nucleus size with age do not decrease lens stiffness in older adult mice. Our work, in addition to prior studies (11, 29, 65), contradict long held hypotheses that the potential causes of age-related lens stiffening are increased lens size and nucleus size (8, 9, 24–28). This study also shows Eph-ephrin signaling contributes to lens shape and normal adult lens growth, and EphA2 is needed for nucleus growth and appropriate GRIN. Further investigation is needed to determine the specific mechanisms for how Eph-ephrin signaling directs lens fiber cell maturation to achieve proper lens shape and sustained lens and nucleus growth.

## Data Availability

The original contributions presented in the study are included in the article/**Supplementary Material**. Further inquiries can be directed to the corresponding author.
