# Supplementary material for: Eph-ephrin signaling affects lens growth and shape, nucleus size, and gradient refractive index in adult mice
Source: Front Ophthalmol (Lausanne). 2025 Oct 31;5:1688964. doi: 10.3389/fopht.2025.1688964 (PMC12615243; doi:10.3389/fopht.2025.1688964)
Supplement: Supplementary file 1 [file Image1.pdf]

Supplementary Material

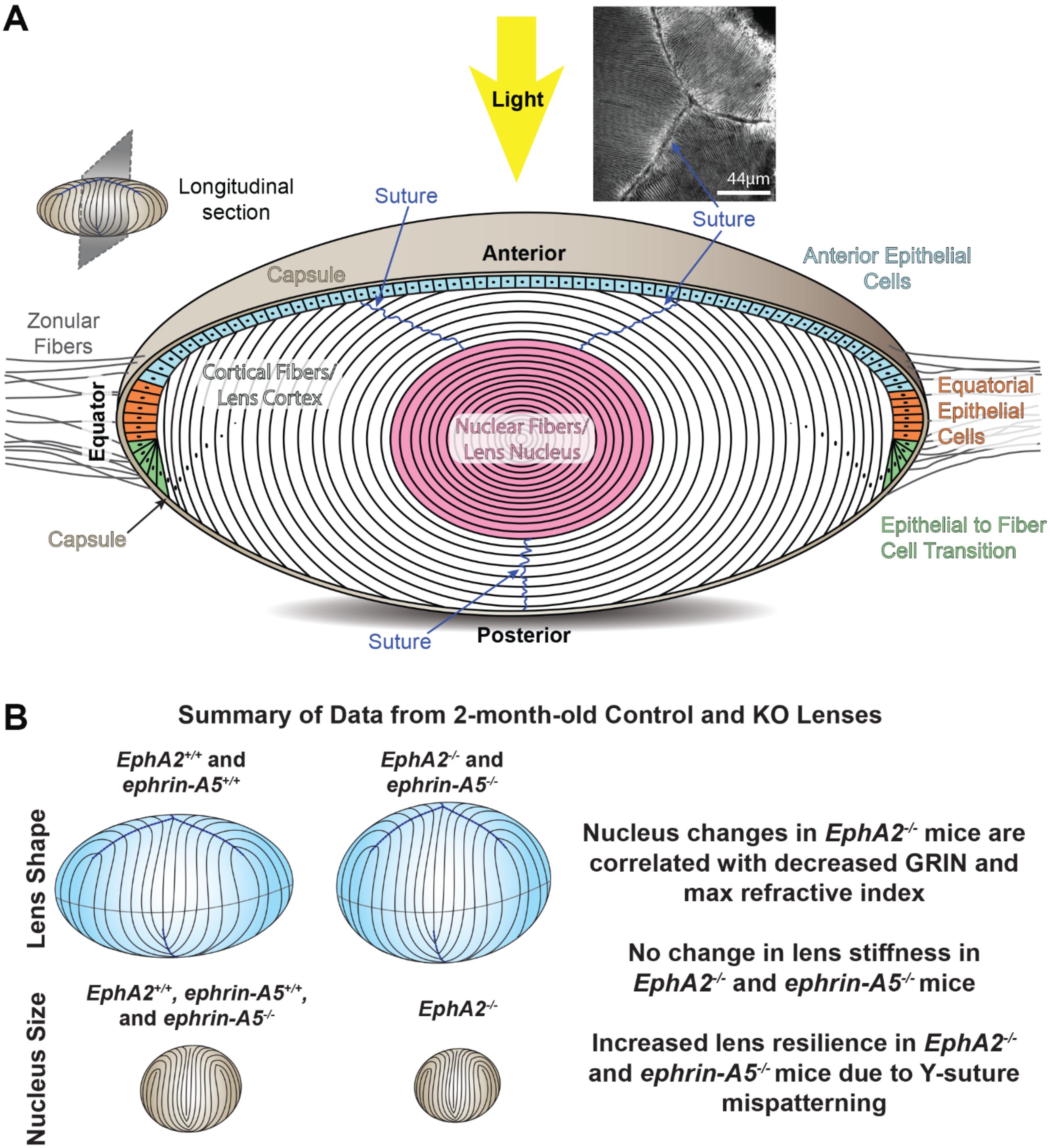

Supplementary Figure 1 – Lens anatomy and previous data summary.

A) A cartoon of lens anatomy through a longitudinal section of the tissue. The lens is encapsulated by a thin collagenous membrane (tan). There are two cell types, a monolayer of epithelial cells (light

blue, orange, and green) covering the anterior hemisphere and a bulk mass of lens fiber cells (white and pink). Anterior epithelial cells (light blue) are quiescent and do not proliferate. At the lens equator, epithelial cells (orange) proliferate and start to differentiate into new generations of fiber cells (green). Fiber cells extend from the anterior to posterior poles, and cell tips meet at the anterior and posterior sutures (dark blue and inset above the cartoon). The lens is suspended in the anterior chamber by zonular fibers attached to the lens capsule and ciliary body. Cartoon is not drawn to scale. Modified from (1). B) A summary of previous morphometrics and biomechanics data from 2-month-old control, *EphA2* knockout (KO or $^{-/-}$) and *ephrin-A5*$^{-/-}$ mice (2, 3). Loss of either EphA2 or ephrin-A5 resulted in more spherical lenses, and lens nuclei were smaller in *EphA2*$^{-/-}$ mice. Change in *EphA2*$^{-/-}$ lens nuclei were correlated with decreased gradient refractive index (GRIN) and max refractive index. There were no obvious changes in lens stiffness in KO lenses, but KO lenses had increased resilience due to Y-suture mispatterning. Cartoon not drawn to scale. Modified from (3).

References

1. Cheng C. Tissue, cellular, and molecular level determinants for eye lens stiffness and elasticity. Front Ophthalmol (Lausanne). 2024;4:1456474.

2. Cheng C. EphA2 and Ephrin-A5 Guide Eye Lens Suture Alignment and Influence Whole Lens Resilience. Invest Ophthalmol Vis Sci. 2021;62(15).

3. Cheng C, Wang K, Hoshino M, Uesugi K, Yagi N, Pierscionek B. EphA2 Affects Development of the Eye Lens Nucleus and the Gradient of Refractive Index. Invest Ophthalmol Vis Sci. 2022;63(1).
